# The First Complete Mitochondrial Genome of the Flathead *Cociella crocodilus* (Scorpaeniformes: Platycephalidae) and the Phylogenetic Relationships within Scorpaeniformes Based on Whole Mitogenomes

**DOI:** 10.3390/genes10070533

**Published:** 2019-07-15

**Authors:** Lei Cui, Rongbo Cao, Yuelei Dong, Xingchen Gao, Jingyi Cen, Songhui Lu

**Affiliations:** 1 Key Laboratory of Eutrophication and Red Tide Prevention, Research Center for Harmful Algae and Marine Biology, Jinan University, Guangzhou 510632, China; 2 Chinese Sturgeon Research Institute, Three Gorges Corporation, Yichang 443100, China

**Keywords:** mitochondrial genome, scorpaeniformes, platycephalidae, *Cociella crocodilus*, phylogenetic analysis

## Abstract

Complete mitochondrial genomes (mitogenomes) are important molecular markers for understanding the phylogenetics of various species. Although recent studies on the mitogenomes of the Scorpaeniformes species have been greatly advanced, information regarding molecular studies and the taxonomic localization of Platycephalidae is still sparse. To further analyze the phylogeny of Platycephalidae, we sequenced the complete mitogenome of *Cociella crocodilus* of the Platycephalidae family within Scorpaeniformes for the first time. The mitogenome was 17,314 bp in length, contained two ribosomal RNA genes (rRNAs), 22 transfer RNA genes (tRNAs), 13 protein-coding genes (PCGs), and two typical noncoding control regions (the control region (CR) and origin of the light strand (OL)). All PCGs used standard initiation codons ATG, apart from cox1. The majority of the tRNA genes could be folded into cloverleaf secondary structures, whereas the secondary structure of tRNASer (AGN) lacked a dihydrouridine (DHU) stem. The CR contained several conserved sequence blocks (CSBs) and eight tandem repeats. In addition, the phylogenetic relationship based on the concatenated nucleotides sequences of 13 PCGs indicated that the Platycephalidae species are relatively basal in the phylogenetic relationships of Scorpaeniformes. Our results may not only advance the origin and the evolution of Scorpaeniformes, but also provide information for the genetic evolution and taxonomy of the teleostean species.

## 1. Introduction

Within the order of Scorpaeniformes, the Platycephalidae family is usually distributed in tropical and temperate coastal or estuarine waters in the Indian and Pacific Oceans. Most of the species live in typical sandy and muddy benthic habitats [1,2]. Platycephalidae, most of which have important commercial and medicinal value [3], consists of approximately 70 valid species of 17 genera [4]. The identification of flatheads usually depends on morphological characteristics such as an elongated body with a spiny depressed head and a large mouth [5]. With the development of molecular biology and molecular genetics, DNA markers (e.g., nuclear and mitochondrial genes) have been widely applied in studies of classification, phylogenetics and genetic diversity of fish. 

The typical mitochondrial genome (mitogenome) is a circular, double-stranded molecule, that ranges in size from 15 kb to 19 kb, and generally contains 37 genes (13 protein-coding genes (PCGs), two ribosomal RNA genes (12S rRNA and 16S rRNA), 22 transfer RNA genes (tRNAs) and two noncoding regions (the control region (CR) and origin of the light strand (O_L_)), and has significant functions in the regulation and initiation of mitogenome transcription and replication [6,7]. In recent years, mitogenomes have been widely used as effective molecular markers for molecular evolution, phylogenetic studies and population genetics [8,9] due to their unique characteristics of maternal inheritance, relatively conserved genome structures, coding content conservation, high mutation rate and low intermolecular genetic recombination level [6]. Based on these advantages, the complete mitogenome of genetic code alteration, gene rearrangement, secondary structures of tRNAs and regions of transcription and replication are also widely utilized for taxonomic phylogenetic analysis [10,11].

To date, complete mitogenomes have been sequenced for approximately 39 Scorpaeniformes species. However, only one complete mitogenome from Platycephalidae, *Platycephalus indicus*, has been sequenced. We previously used the mitochondrial genome to study the genetic relationships of Scorpaeniformes [12], which does not include the Platycephalidae family, although phylogenetic trees based on nuclear genes and partial mitochondrial gene sequences were constructed to analyze interspecies relationships with Scorpaeniformes in previous research. Nevertheless, the difference between whether flatheads or scorpionfishes are ‘the most basal clade’ of Scorpaeniformes was studied [13,14,15]. The markers of small fragments may not provide sufficient evidence to explain phylogenetic relationships. To research higher-level relationships among Scorpaeniformes, the complete mitogenome of one Platycephalidae species, *Cociella crocodilus*, was sequenced in our study and we analyzed the gene content and organization compared with other Scorpaeniformes species. In addition, the phylogenetic tree based on the thirteen PCG sequences was reconstructed by Maximum Likelihood (ML) and Bayesian inference (BI) methods to understand the higher phylogeny of Scorpaeniformes. 

## 2. Methods

### 2.1. Sampling and DNA Extraction

A specimen of *C. crocodilus* was captured in the Pearl River estuary (N 21°45′, E 133°36′), China, in July 2016.The field study did not involve endangered or protected species, in accordance with the IUCN Red List. The research was approved by the Institutional Animal Care and Use Committee at Jinan University, with no ethical code associated. The experiments were conducted in accordance with international guidelines for the care and treatment of laboratory animals. The samples were identified according to morphological characteristics and were then conserved in 95% ethanol and stored at −80 °C until processing. Dorsal muscle tissue was removed to extract the total genomic DNA using the Animal Tissue Genomic DNA Extraction Kit (SangonBiotech, Shanghai, China) according to the manufacturer’s instructions. The extracted DNA was used to amplify the complete mitogenome of *C. crocodilus* by polymerase chain reaction (PCR).

### 2.2. Polymerase Chain Reaction Amplification and Sequencing

To obtain the complete *C. crocodilus* mitogenome sequence, PCR amplification was performed with several primer pairs designed based on aligned mitogenome sequences of *Chelidonichthys kumu* (GenBank: KY379222) and *Lepidotrigla microptera* (GenBank: KY012348.1) (Table S1) [12]. All PCR reactions were conducted with LA Taq DNA polymerase using Premix LA Taq (Takara, Dalian, China) according to the following cycling conditions: beginning with an initial denaturation step at 95 °C for 1 min, followed by 35 cycles of denaturation at 95 °C for 20 s, annealing at 55 °C for 45 s and elongation at 72 °C for 1–5 min depending on the length of the segment. The sequences of the PCR-amplified products were determined on a 3730XL DNA Analyzer (Beijing Genomics Institute, Shenzhen China). 

### 2.3. Sequence Analysis

In order to obtain the final complete sequence, the obtained sequenced fragments were assembled through the program Seqman within the Lasergene software and then manually checked [16]. Sequence annotation was performed using NCBI BLAST (http://blast.ncbi.nlm.nih.gov/Blast) and the DNASTAR package (DNASTAR Inc, Madison, WI, USA). The tRNA genes were identified using the default search mode of the tRNAscan-SE program [17]. Secondary structures of tRNA genes and O_L_ were inferred using the software RNAstructure [18] with vertebrate mitochondrial predictors. The Dual Organellar GenoMe Annotator (DOGMA) automated the annotation of two rRNA and 13 PCGs [19]. The codon usage of the protein-coding genes and the nucleotide composition were determined using MEGA 6.0 [20]. The tandem repeats in the control region were predicted using the Tandem Repeats Finder available online [21]. The formulas AT-skew [(A − T)/(A + T)] and GC-skew [(G − C)/(G + C)] were used to measure the nucleotide composition skewness [22]. The final mitogenome sequence of the *C. crocodilus* was submitted to the GenBank database under the accession number MH521260.1.

### 2.4. Phylogenetic Analysis

Based on 39 Scorpaeniformes mitogenomes available in the GenBank, phylogenetic analysis was performed (Table 1). Three outgroups taxa were assigned, i.e., Perciformes fishes (*Percalates novemaculeata* (NC_024850.1) and *Siniperca knerii* (NC_015987.1) [23,24]. The concatenated nucleotide sequence alignment was used in IQ-TREE and MrBayes v 3.2.4 to determine maximum likelihood (ML) and Bayesian inference (BI) for phylogenetic analysis [25,26]. All the sequences of the genes were aligned individually by ClustalX [27]. GTR+F+R6 was selected as the appropriate model for the nucleotide sequences by ModelFinder in IQ-TREE based on Akaike’s information criterion (AIC) [25,28]. Four independent Markov chains were simultaneously used at 1,000,000 generations with sampling every 1000 generations for BI analyses. The first 25% were discarded as burn-in. The BI Tree was considered to be reached since the average standard deviation of the split frequencies was below 0.01. ML analysis was carried out with 100 bootstrap replicates based on the default parameters. 

## 3. Results and Discussion 

### 3.1. Mitogenome Organization, Structure and Skewness

The complete mitogenome of *C. crocodilus* formed a closed circular molecule with a total length of 17,314 bp in size, which was the largest of all the sequenced Scorpaeniformes species available in the GenBank to date. The mitogenome contained 37 genes identical to other Scorpaeniformes species: two rRNA genes (12S rRNA and 16S rRNA), 22 tRNAs (one for each amino acid, two for Leucine and Serine), 13 PCGs (*cox1-3, nad1-6, nad4L, atp6, atp8 and cytb*) and two noncoding regions (control region (CR) and O_L_) (Figure S1 and Table 2). Except *ND6* and eight tRNA genes (Gln, Ala, Asn, Cys, Tyr, Ser (UCN), Glu and Pro), which were distributed on the light strand (L-strand), most of the other mitochondrial genes were distributed on the heavy strand (H-strand). The mitogenome of *C. crocodilus* had a total overlap of 29 bp (ranging from 1 to 10 bp) between genes at nine locations, among which *atp8*- *atp6* (10 bp) and *nad4L*-*nad4* (7 bp) often occurred in the Metazoa. A total of 162 bp nucleotides ranged from 1 to 79 bp in 15 spacers. The longest 79 bp spacer was between tRNA^L1^ and *nad1* (Table 2). The nucleotide composition of the *C*. *crocodilus* is as follows: A = 29.14%, T = 27.19%, G = 15.91% and C = 27.76%, which shows a high A+T content of 56.3%. The A+T content is higher than most sequenced Scorpaeniformes species, such as *Sebates schlegeli* (53.8%), *Scorpaenopsis cirrosa* (54.27%), and *Hexagrammos lagocephalus* (53.27%). The exception is *Synanceia verrucosa* (59.35%). The fact that the A + T content was higher than the G+C content is in accordance with the characteristics of the mitogenome of metazoan animals [29]. For the complete mitogenome sequence of *Cociella crocodilus*, the AT skew was positive (0.0348) and the GC skew was negative (−0.2713). Generally, the teleostean mitogenomes tend to have identical positive AT-skew and negative GC-skew. All of the GC skew was negative among Scorpaeniformes species and most of the AT skew was positive, apart from *Satyrichthys amiscus* (−0.0058), *Pleurogrammus monopterygius* (−0.0012), *Cottus hangiongensis* (−0.0011), and *Cottus poecilopus* (−0.0075) [30,31].

### 3.2. Protein-Coding Genes 

**Figure 1:**
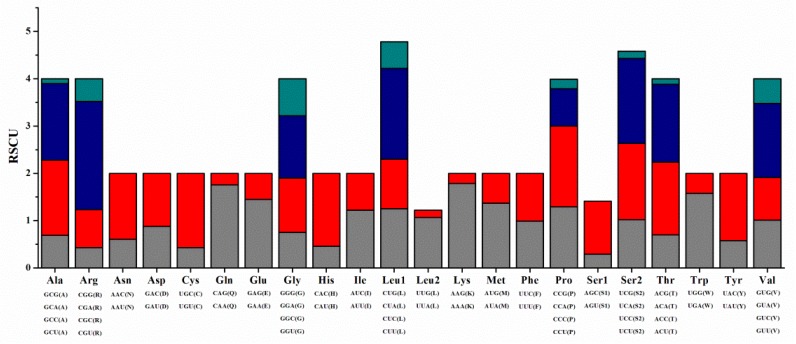
Relative synonymous codon usage (RSCU) of the *C. crocodilus* mitogenome.

**Figure 2:**
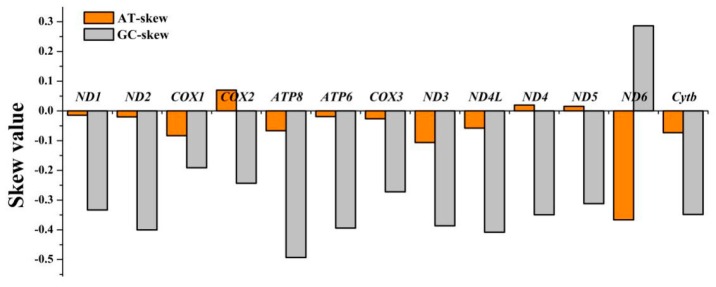
The AT skew and GC skew in the PCGs of the *C. crocodilus* mitogenome.

The complete mitogenome of *C. crocodilus* had 13 PCGs with a total length of 11,445 bp, 12 of which utilize ATG for the start codon, whereas *cox1* utilizes GTG. In teleost mitogenomes, initiator codons are often used selectively (e.g., *cox1* of the whiting, *Merlangius merlangus*; *cox1* of the haddock, *Melanogrammus aeglefinus*; and *cox1* and *nad4l* of the Globose Head Whiptail, *Cetonurus globiceps*) [32,33]. Eight of the 13 PCGs (*nad1, nad2, cox1, atp8, atp6, cox3, nad4l,* and *nad5*) used the typical stop codon TAA. The two genes *nad3* and *nad6* were terminated with TAG. The incomplete termination codon T was used by *cox2, nad4*, and *cytb* (Table 2). Such incomplete termination codons were due to the 3’ end of the mRNA appearing to have posttranscriptional polyadenylation [34]. Except for the AGA and AGG termination codons, the total number of codons in the PCGs of *C*. *crocodilus* was 3,804, among which three amino acids (Leu1, 513, Ala, 353, and Thr, 302) were the most frequent (Figure 1 and Table S2). In addition to the three genes of *C. crocodilus*, *cox2, nad4* and *nad5*, most of the AT skew was negative. Most of the GC skew values were slightly negative, except for *nad6*, indicating that there were more Ts and Cs in most PCGs, which is consistent with previous research (Figure 2).

### 3.3. Transfer RNA Genes and Ribosomal RNA Genes

There were 22 tRNA genes ranging from 67 bp (trnP) to 76 bp (trnL1) identified in the *C. crocodilus* mitogenome. Among them, 14 tRNA genes were encoded on the H-strand, and the other eight tRNA genes were coded by the L-strand (Table 2). Most of the tRNAs were folded into an ordinary cloverleaf secondary structure (Figure S2), except trnS2 (AUN), which lacked a DHU stem. This phenomenon is identical to other mitogenomes of teleost fishes, including the Scorpaeniformes species [12,35]. The standard structure of the amino acid acceptor stem was 7 bp, while trnaV, trnF, and trnaL1 (UUR) were 9 bp. Further, there were 24 base-pair mismatches present in the tRNA secondary structures, all of which formed a weak bond (G-U pairs). The compositions of the complete sequences of all 22 tRNAs showed both slightly positive AT skew (0.0136) and GC skew (0.0473), indicating that tRNAs biased toward As and Gs. The 12S and 16S rRNA genes were 1,021 bp and 1,713 bp in length, respectively, and similarly, the AT skew (0.0763 and 0.2227) was slightly positive and the GC skew (−0.1952 and −0.1064) was negative. They were situated between trnF and trnL1 and there is a trnV between rRNAs similar to other vertebrate mitogenomes to date [36,37,38]. 

### 3.4. Noncoding Regions

There were two long noncoding regions in the mitogenome of *C. crocodilus*, O_L_ and CR, which were closely related to the initiation of the replication and transcription of the mitogenome [39,40]. Located between trnN and trnC with a length of 30 bp, the relatively short gene O_L_ was folded into a hairpin secondary structure (Figure S3). The CR was the longest noncoding region and extended over 1447 bp between trnP and trnF, with a 68.76% A + T content, which is the highest among Scorpaeniformes species to date. Similarly to other teleost fish, there were several conserved sequence blocks (CSBs) identified in CR, which were shown to play indispensable roles in mitochondrial metabolism [41]. The central conserved domain contained blocks CSB-D, -E and -F, while the typical conserved sequence block region involved blocks CSB-1, CSB-2 and CSB-3 (Figure S4). Consistent with other Scorpaeniformes species, the conserved motifs ATGTA and its complement TACAT as the recognition sites are commonly found in the CR of teleosts. In addition, eight concatenated repeats in *C. crocodilus* were confirmed with the Tandem Repeats Finder program [21]. This phenomenon occurs only in *Enophrys diceraus* and *Sebastiscus marmoratus*, but never in other Scorpaeniformes species [42,43]. These tandem repeats may be one reason why the *C. crocodilus* mitogenome was bigger than in other sequenced Scorpaeniformes species.

### 3.5. Phylogenetic Relationships

**Figure 3:**
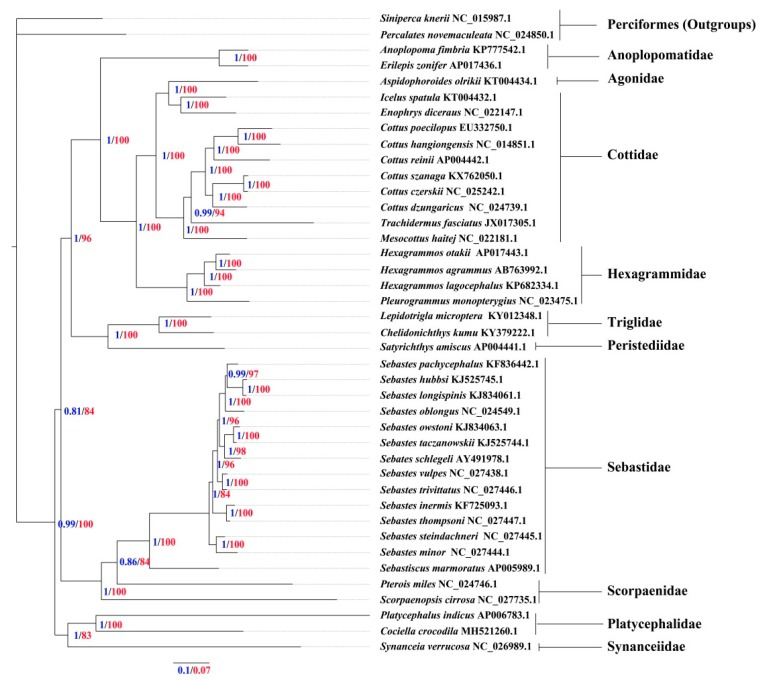
Phylogenetic tree based on the nucleotide sequences of the 13 PCGs, using Bayesian inference (BI) and maximum likelihood (ML) methods, among Scorpaeniformes species. Perciformes fishes (*P. novemaculeata* (NC_024850.1) and *S. knerii* (NC_015987.1)) were used as outgroups. Numbers above the branches refer to Bayesian posterior probability values (blue numbers) and ML bootstrap values (red numbers).

To determine the phylogenetic position of *C. crocodilus*, 39 Scorpaeniformes species’ mitogenomes were obtained from the GenBank and the mitogenomes of Perciformes fishes (*P. novemaculeata* (NC_024850.1) and *S. knerii* (NC_015987.1) were used for outgroups (Figure 3). Phylogenetic trees inferred from the ML and BI methods were constructed for the nucleotide sequences of 13 PCGs. The results clearly indicate that flatheads, scorpionfishes and rockfishes together comprised Scorpaeniformes as a paraphyletic group. The phylogenetic trees revealed three strongly supported clades: (I) Synanceiidae and Platycephalidae, (II) Scorpaenidae and Sebastidae, and (III) Anoplopomatidae, Agonidae, Triglidae, Peristediidae, Hexagrammidae and Cottidae. The best supported phylogenetic relationships of Scorpaeniformes are as follows: (Synanceiidae + (Platycephalidae + ((Scorpaenidae + Sebastidae) + ((Triglidae + Peristediidae) + (Agonidae + (Hexagrammidae + (Anoplopomatidae + Cottidae))))))). There was a conflict with our work based on complete mitogenomes [12]. In a previous study, Synanceiidae was as a sister to Scorpaenidae + Sebastidae. The phylogenetic analysis results were similar to earlier studies (although only Synanceiidae, Scorpaenidae and Sebastidae were available) [44]. Based on the results of the current study, Synanceiidae was proposed to be one of the most basal taxa in the order of Scorpaeniformes after adding new taxa (Anoplopomatidae, Agonidae and Platycephalidae) to the phylogenetic analysis. As the only sequenced mitogenome to date, the Synanceiidae family requires further phylogenetic research. *C. crocodilus*, within Platycephalidae, which branches into an independent sub-branch, shares a close ancestry with *P. indicus*. The topology data obtained in this study indicates that the flatheads were more basal than scorpionfishes among Scorpaeniformes, which is not consistent with previous studies involving phylogenetic analysis on the basis of segmental mitogenomes (16S rRNA) and nuclear genes (*rag1*, *rag2*, and *rh*) [13]. Whether the difference in the phylogenetic analysis is due to introgression, hybridization and lineage sorting is unknown. However, some phylogenetic results also showed that flatheads were relatively basal species based on a modified time-calibrated model [14,15]. Additional research should be conducted to discover other un-sequenced families and employ other molecular datasets to elucidate phylogenetic relationships among Scorpaeniformes species.

## Figures and Tables

**Table 1:** Summary of the base composition and skewness of whole mitochondrial genomes and 13 protein-coding genes (PCGs) among 39 Scorpaeniformes species.

Family	Species	Accession Number	Size (bp)	Whole Genome Composition							PCGs	
				A%	G%	T%	C%	A + T%	AT skew	GC skew	AT skew	GC skew
Anoplopomatidae	*Erilepis zonifer*	AP017436.1	16,500	26.70	17.79	29.03	26.48	53.18	0.0041	−0.2400	−0.0965	−0.2715
Anoplopomatidae	*Anoplopoma fimbria*	KP777542.1	16,507	26.07	18.34	29.58	26.01	52.08	0.0010	−0.2346	−0.0994	−0.2701
Agonidae	*Aspidophoroides olrikii*	KT004434.1	17,200	27.30	16.90	27.28	28.49	55.80	−0.0214	−0.2350	−0.1153	−0.2662
Cottidae	*Icelus spatula*	KT004432.1	16,384	26.43	17.43	30.04	26.03	52.46	0.0078	−0.2656	−0.0987	−0.2935
Cottidae	*Trachidermus fasciatus*	JX017305.1	16,536	26.33	18.13	30.07	25.47	51.80	0.0167	−0.2478	−0.0815	−0.2750
Cottidae	*Cottus poecilopus*	EU332750.1	16,560	25.69	18.18	30.39	25.74	51.43	−0.0011	−0.2513	−0.1040	−0.2822
Cottidae	*Cottus hangiongensis*	NC_014851.1	16,598	25.48	18.22	30.40	25.87	51.36	−0.0075	−0.2506	−0.1135	−0.2840
Cottidae	*Enophrys diceraus*	NC_022147.1	16,976	27.53	16.65	28.64	27.19	54.71	0.0062	−0.2648	−0.0869	−0.2987
Cottidae	*Cottus reinii*	AP004442.1	16,561	26.30	17.63	30.28	25.78	52.09	0.0100	−0.2640	−0.0919	−0.2999
Cottidae	*Mesocottus haitej*	NC_022181.1	16,527	26.64	17.35	29.88	26.12	52.76	0.0099	−0.2653	−0.0853	−0.2995
Cottidae	*Cottus szanaga*	KX762050.1	16,518	26.51	17.39	29.94	26.16	52.67	0.0067	−0.2650	−0.0853	−0.3000
Cottidae	*Cottus czerskii*	NC_025242.1	16,534	26.40	17.47	30.07	26.07	52.47	0.0063	−0.2651	−0.0850	−0.3130
Cottidae	*Cottus dzungaricus*	NC_024739.1	16,527	26.93	17.07	29.70	26.30	53.23	0.0119	−0.2701	−0.0822	−0.3135
Hexagrammidae	*Pleurogrammus monopterygius*	NC_023475.1	16,575	27.05	17.15	28.68	27.12	54.17	−0.0012	−0.2517	−0.0956	−0.2913
Hexagrammidae	*Hexagrammos lagocephalus*	KP682334.1	16,505	26.98	17.26	29.48	26.29	53.27	0.0130	−0.2615	−0.0836	−0.3002
Hexagrammidae	*Hexagrammos otakii*	AP017443.1	16,513	26.90	17.33	29.87	25.90	52.80	0.0189	−0.2656	−0.0727	−0.3067
Hexagrammidae	*Hexagrammos agrammus*	AB763992.1	16,514	26.88	17.23	29.72	26.15	53.03	0.0137	−0.2659	−0.0839	−0.3009
Peristediidae	*Satyrichthys amiscus*	AP004441.1	16,526	27.06	17.00	28.56	27.38	54.44	−0.0058	−0.2537	−0.1030	−0.2883
Platycephalidae	*Platycephalus indicus*	AP006783.1	16,542	27.32	16.99	30.12	25.57	52.88	0.0332	−0.2787	−0.0537	−0.3143
Platycephalidae	*Cociella crocodila*	MH521260.1	17,314	29.14	15.91	27.76	27.19	56.33	0.0348	−0.2713	−0.0375	−0.2911
Scorpaenidae	*Pterois miles*	NC_024746.1	16,497	27.40	18.26	28.74	25.60	53.00	0.0340	−0.2229	−0.0509	−0.2420
Scorpaenidae	*Scorpaenopsis cirrosa*	NC_027735.1	16,966	27.91	17.71	28.02	26.35	54.27	0.0288	−0.2254	−0.0601	−0.2448
Sebastidae	*Sebastes oblongus*	NC_024549.1	16,396	27.91	16.96	28.72	26.41	54.32	0.0276	−0.2574	−0.0595	−0.2842
Sebastidae	*Sebastes thompsoni*	NC_027447.1	16,405	27.98	17.04	28.21	26.77	54.75	0.0222	−0.2468	−0.0677	−0.2781
Sebastidae	*Sebastes minor*	NC_027444.1	16,408	27.79	17.33	27.63	27.25	55.04	0.0099	−0.2292	−0.0815	−0.2609
Sebastidae	*Sebastes trivittatus*	NC_027446.1	16,409	27.86	17.09	28.37	26.67	54.54	0.0218	−0.2480	−0.0690	−0.2789
Sebastidae	*Sebastes taczanowskii*	KJ525744.1	16,452	27.71	17.29	28.53	26.47	54.18	0.0229	−0.2452	−0.0709	−0.2748
Sebastidae	*Sebastes owstoni*	KJ834063.1	16,465	27.71	17.30	28.41	26.57	54.28	0.0210	−0.2430	−0.0748	−0.2723
Sebastidae	*Sebates schlegeli*	AY491978.1	16,525	27.47	17.45	28.74	26.34	53.80	0.0210	−0.2444	−0.0680	−0.2742
Sebastidae	*Sebastiscus marmoratus*	AP005989.1	17,301	28.69	16.51	28.14	26.67	55.36	0.0364	−0.2605	−0.0592	−0.2855
Sebastidae	*Sebastes pachycephalus*	KF836442.1	16,415	27.77	17.24	28.66	26.33	54.10	0.0266	−0.2488	−0.0635	−0.2786
Sebastidae	*Sebastes longispinis*	KJ834061.1	16,445	27.91	17.12	28.31	26.66	54.57	0.0230	−0.2462	−0.0699	−0.2713
Sebastidae	*Sebastes steindachneri*	NC_027445.1	16,450	27.36	17.54	28.01	27.09	54.46	0.0049	−0.2298	−0.0873	−0.2587
Sebastidae	*Sebastes hubbsi*	KJ525745.1	16,453	27.86	17.20	28.28	26.66	54.52	0.0221	−0.2436	−0.0671	−0.2714
Sebastidae	*Sebastes vulpes*	NC_027438.1	16,462	27.71	17.14	28.61	26.55	54.25	0.0214	−0.2506	−0.0706	−0.2808
Sebastidae	*Sebastes inermis*	KF725093.1	16,504	27.77	17.13	28.34	26.76	54.53	0.0186	−0.2466	−0.0714	−0.2735
Synanceiidae	*Synanceia verrucosa*	NC_026989.1	16,506	31.01	15.06	25.60	28.34	59.35	0.0451	−0.2593	−0.0286	−0.2883
Triglidae	*Chelidonichthys kumu*	KY379222.1	16,495	26.63	17.04	31.13	25.20	51.83	0.0277	−0.2925	−0.0640	−0.3351
Triglidae	*Lepidotrigla microptera*	KY012348.1	16,610	26.53	17.15	31.28	25.05	51.58	0.0286	−0.2918	−0.0666	−0.3326

**Table 2:** Organization constituents of the mitochondrial genome of *Cociella crocodilus*.

Feature	Strand *	Position	Spacer (+)/Overlap (−)	Start/Stop codon
tRNA-Phe (F)	H	1–67	0	
12S rRNA	H	68–1088	0	
tRNA-Val (V)	H	1089–1160	−2	
16S rRNA	H	1161–2873	79	
tRNA-Leu (L1)	H	2872–2947	3	
*nad1*	H	3027–4001	−1	ATG/TAA
tRNA-Ile (I)	H	4005–4074	23	
tRNA-Gln (Q)	L	4074–4144	1	
tRNA-Met (M)	H	4168–4237	6	
*nad*2	H	4239–5288	0	ATG/TAA
tRNA-Trp (W)	H	5295–5366	1	
tRNA-Ala (A)	L	5367–5435	30	
tRNA-Asn (N)	L	5437–5509	0	
tRNA-Cys (C)	L	5540–5607	1	
tRNA-Tyr (Y)	L	5608–5675	0	
*cox*1	H	5677–7227	3	GTG/TAA
tRNA-Ser (S1)	L	7228–7298	5	
tRNA-Asp (D)	H	7302–7374	0	
*cox*2	H	7380–8070	1	ATG/T
tRNA-Lys (K)	H	8071–8145	−10	
*atp* 8	H	8147–8311	−1	ATG/TAA
*atp* 6	H	8302–8985	−1	ATG/TAA
*cox*3	H	8985–9770	0	ATG/TAA
tRNA-Gly (G)	H	9770–9841	−2	
*nad*3	H	9842–10,192	0	ATG/TAG
tRNA-Arg (R)	H	10,191–10,259	−7	
*nad*4l	H	10,260–10,556	0	ATG/TAA
*nad*4	H	10,550–11,930	−7	ATG/T
tRNA-His (H)	H	11,931–11,999	0	
tRNA-Ser (S1)	H	12,002–12,069	2	
tRNA-Leu (L1)	H	12,074–12,146	4	
*nad*5	H	12,147–13,985	0	ATG/TAA
*nad*6	L	13,982–14,503	−4	ATG/TAG
tRNAGlu (E)	L	14,505–14,573	1	
*cytb*	H	14,576–15,725	2	ATG/T
tRNA-Thr (T)	H	15,726–15,797	0	
tRNA-Pro (P)	L	15,797–15,867	−1	
Control region	H	15,868–17,314	0	

* H and L refer to the heavy and light strand, respectively.

## Supplementary Materials

The following are available online at https://www.mdpi.com/2073-4425/10/7/533/s1, Figure S1: Organization of the complete mitochondrial genome of *C. crocodilus*, Figure S2: Predicted secondary structures of 22 tRNA genes in the *C. crocodilus* mitogenome. Figure S3: The putative secondary hairpin structural features of the O_L_ in the mitogenome of *C. crocodilus*, Figure S4: The structure of the control region in the mitogenome of *C. crocodilus*, Table S1: Primer pairs used for PCR amplification of *C. crocodilus* mitogenome, Table S2: Codon number and RSCU in *C. crocodilus* mitochondrial PCGs.
