# Supplementary material for: The First Complete Mitochondrial Genome of the Flathead Cociella crocodilus (Scorpaeniformes: Platycephalidae) and the Phylogenetic Relationships within Scorpaeniformes Based on Whole Mitogenomes
Source: Genes (Basel). 2019 Jul 15;10(7):533. doi: 10.3390/genes10070533 (PMC6678826; doi:10.3390/genes10070533)
Supplement: Supplementary file 1 [file genes-10-00533-s001.pdf]

S1 Table. Primer pairs used for PCR amplification of *C. crocodilus* mitogenome.

Primer	Sequences(*H. quoyi*)	Annealing temperature(℃)
1F	CGGCGTAAAGCGTGGTTA	55
1R	GTTGGCGATAAGAACTCAAAAGAG	
2F	GAACTGAGCCAACCAGGAGC	55
2R	GCTTTCGTGGGTTCAGTATCAT	
3F	TTTAACATTCCGCCCTGTCTC	55
3R	GGAGGGATAGGAGAAGTAGAACG	
4F	ATGATACGGACGAGCAGACG	55
4R	CCAATAACAATAAACGGGTCCTC	
5F	CTCTTTTACCGCCATCTACAGC	55
5R	CGAGACAGAGGCAAATGGTG	
6F	TCCCTAATAAACTGGGAGGTGTC	55
6R	TTGGAAAAGTTATGCGTGGG	

S2 Table. Codon number and RSCU in *C. crocodilus* mitochondrial PCGs. A total of 3,804 codons were analysed excluding the initiation and termination codons. Amino acids encoded by these codons are labelled according to the IUPAC-IUB single-letter amino acid codes.

**Codon**	**n(RSCU)**	**Codon**	**n(RSCU)**	**Codon**	**n(RSCU)**	**Codon**	**n(RSCU)**
UCU(S2)	42(1.02)	UAU(Y)	32(0.58)	UGU(C)	5(0.43)	UCU(S2)	42(1.02)
UCC(S2)	66(1.61)	UAC(Y)	78(1.42)	UGC(C)	18(1.57)	UCC(S2)	66(1.61)
UCA(S2)	74(1.8)	UAA(*)	0(0)	UGA(W)	93(1.58)	UCA(S2)	74(1.8)
UCG(S2)	6(0.15)	UAG(*)	1(2)	UGG(W)	25(0.42)	UCG(S2)	6(0.15)
CCU(P)	70(1.29)	CAU(H)	24(0.46)	CGU(R)	8(0.43)	CCU(P)	70(1.29)
CCC(P)	93(1.71)	CAC(H)	80(1.54)	CGC(R)	15(0.8)	CCC(P)	93(1.71)
CCA(P)	43(0.79)	CAA(Q)	80(1.76)	CGA(R)	43(2.29)	CCA(P)	43(0.79)
CCG(P)	11(0.2)	CAG(Q)	11(0.24)	CGG(R)	9(0.48)	CCG(P)	11(0.2)
ACU(T)	53(0.7)	AAU(N)	38(0.61)	AGU(S1)	12(0.29)	ACU(T)	53(0.7)
ACC(T)	116(1.54)	AAC(N)	86(1.39)	AGC(S1)	46(1.12)	ACC(T)	116(1.54)
ACA(T)	124(1.64)	AAA(K)	67(1.79)	AGA(S1)	0(0)	ACA(T)	124(1.64)
ACG(T)	9(0.12)	AAG(K)	8(0.21)	AGG(S1)	0(0)	ACG(T)	9(0.12)
GCU(A)	61(0.69)	GAU(D)	34(0.88)	GGU(G)	44(0.75)	GCU(A)	61(0.69)
GCC(A)	140(1.59)	GAC(D)	43(1.12)	GGC(G)	68(1.15)	GCC(A)	140(1.59)
GCA(A)	143(1.62)	GAA(E)	73(1.45)	GGA(G)	78(1.32)	GCA(A)	143(1.62)
GCG(A)	9(0.1)	GAG(E)	28(0.55)	GGG(G)	46(0.78)	GCG(A)	9(0.1)

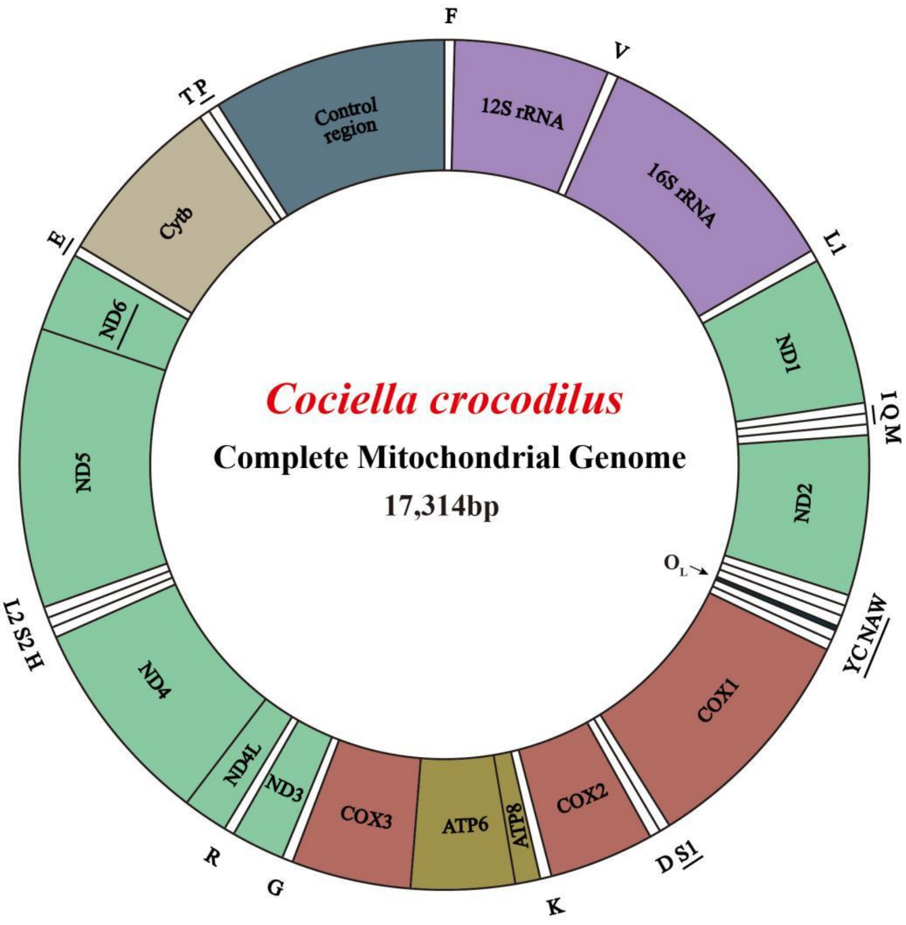

Fig. S1 Organization of the complete mitochondrial genome of *C. crocodilus.* Single letter amino acids representing tRNAs refer to IUPAC-IUB (S1:trnL^{AGN}; S2:trnL^{UCN}; L1:trnL^{CUN}; L2: trnL^{UUR}). The coding gene name on the heavy strand is not underlined, while the coding gene on the light chain is underlined.

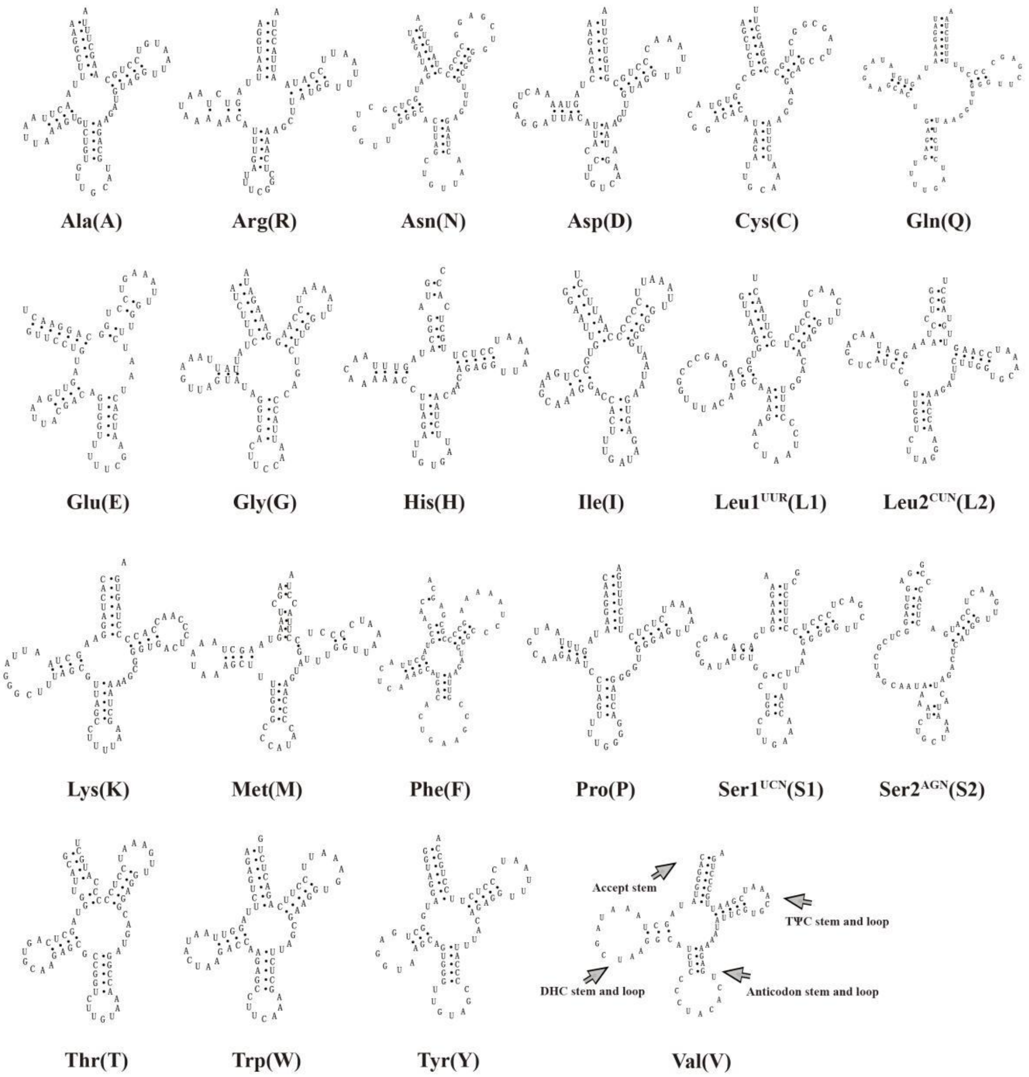

Fig. S2 Predicted secondary structures of 22 tRNA genes in the *C. crocodilus* mitogenome.

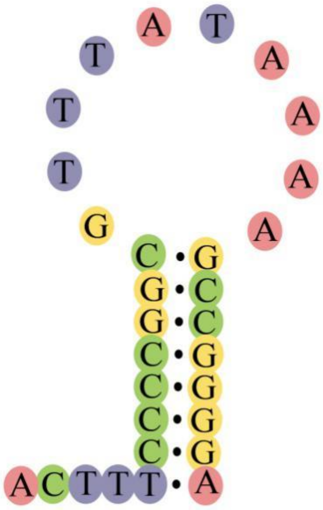

Fig. S3 The putative secondary hairpin structural features of the O_L in the mitogenome of *C. crocodilus*.

trnaP-15,868AATTCTTATATATACATATATGTATTTTCACCATAAATTTATTTTAACCTTACA

AGGAACATGTTCGAATAAGATATACATATGATTTATTTGACATAAATTTAAAATTCCAATA

AAGGGTCTATTAAATACTCCACAATAAGGTAGTTAAATAATTTCATACCTTGTCTCACAT

CBS-F

TCCATTAAATAAATAATATAATTAGCAGTAAGAACCGACCATAAAACTATACCTTAATGC

CBS-E

CAACGGTTATTGAAGGTGAGGGACAAAAACTGTGGGGGTTTCACTTCATGAACTATTC

CBS-D

CTGGCATTTGGTTCCTACTTCAGGGCCATTAATTGGTATCATTCCTCATACTTTCATTGAC

GCTTGCATAAGTTAATGGTGGTACACATGTCTGGGAGCACCCAGCATCCTAATTATAGG

TGCATAGGGTTCTCTTTTTTCTTTATCCTTTCACTTACATTTCACAGTGCAAAGTAATATG

ATATAACAAGGTTGAACATTTTTTCTTGCTTGAAATAAATCTTACTGAATTATATTAGAAT

CBS-1

ATCTTTTAATAATTGCATAACTGATTTCAAGTGCATAAGTTATACATACTATTCTTTATCTC

CBS-2

CCCCGGCTTCCGGGCGAAAACCCCCCCTACCCCCCCAACTACTAAAGTTTCTTAATGAT

CBS-3

CCTACAAACCCCCCGGAAATAGGAAAAACCTTAAATAGTTCACTCACCTTCTACCATGT

ATACGAACATTAGTAAAGTCGAACATGAGAATGATGGGGTATAATAAAAATTATATCTAT

Repeat 1

TAATATATAGTATTTATTTATGCTTATATATCCTCATAAACACAACCGCCCTGATAAACCTT

Repeat 2

AAGTTCCATAAACATATACCTTTTATATAGTATTATATATCCTCATAAACACAACCGCCCT

Repeat 3

GATAAACCTTAAGTTCCATAAACATATACCTTTTATATAGTATTATATATCCTCATAAACAC

Repeat 4

AACCGCCCTGATAAACCTTAAGTTCCATAAACATATACCTTTTATATAGTATTATATATCCT

CATAAACACAACCGCCCTGATAAACCTTAAGTTCCATAAACATATACCTTTTATATAGTAT

Repeat 5

TATATATCCTCATAAACACAACCGCCCTGATAAACCTTAAGTTCCATAAACATATACCTTT

Repeat 6

TATATAGTATTATATATCCTCATAAACACAACCGCCCTGATAAACCTTAAGTTCCATAAAC

Repeat 7

ATATACCTTTTATATAGTATTATATATCCTCATAAACACAACCGCCCTGATAAACCTTAAG

Repeat 8

TTCCATAAACATATACCTTTTATATAGTATTATATATCCTCATAAACACAACCGCCCTGATA

Fig. S4 The structure of the control region in mitogenome of *C. crocodilus*. The sequences marked gray are conserved motifs ATGTA and its complement TACAT. The green and purple background denote the eight tandem repeats.
